# Æsculapian Society of the Wabash Valley

**Published:** 1875-06-15

**Authors:** 


ÆSCULAPIAN SOCIETY OF THE WABASH VALLEY.—This Society held a regular and interesting meeting in Terre Haute, Indiana, on the 26th and 27th ults.

